# Supplementary material for: Signatures of magnetism control by flow of angular momentum
Source: Nature. 2024 Sep 4;633(8030):548–53. doi: 10.1038/s41586-024-07914-y (PMC11410660; doi:10.1038/s41586-024-07914-y)
Supplement: Supplementary file 1 — This file contains Supplementary Notes 1–4, Supplementary Figs. 1–11, Supplementary Tables 1 and 2 as well as Supplementary References. [file 41586_2024_7914_MOESM1_ESM.pdf]

nature portfolio

https://doi.org/10.1038/s41586-024-07914-y

Supplementary information

Signatures of magnetism control by flow of angular momentum

In the format provided by the authors and unedited

Supplementary Material for

Signatures of magnetism control by flow of angular momentum

L. Chen[1], Y. Sun[1], S. Mankovsky[2], T. N. G. Meier[1], M. Kronseder[3], C. Sun[6,7], A. Orekhov[6], H. Ebert[2], D. Weiss[3] and C. H. Back[1,4,5]

[1]*Department of Physics, Technical University of Munich, Munich, Germany*
[2]*Department of Chemistry, Ludwig Maximilian University, Munich, Germany*
[3]*Institute of Experimental and Applied Physics, University of Regensburg, Regensburg, Germany*
[4]*Munich Center for Quantum Science and Technology, Munich, Germany*
[5]*Center for Quantum Engineering, Technical University of Munich, Munich, Germany*
[6]*Department of Chemistry, Technical University of Munich, Munich, Germany*
[7]*TUMint.Energy Research GmbH, Department of Chemistry, Technical University of Munich, Munich, Germany*

Table of Contents:

1. Characterization of the Pt/Al/Fe/GaAs multilayers

2. Ruling out possible detrimental effects arising from the application of a dc current

3. Discussion of the effect of the magnetic proximity effect

4. Theoretical calculation of the modification of magnetic moment and magnetic anisotropy

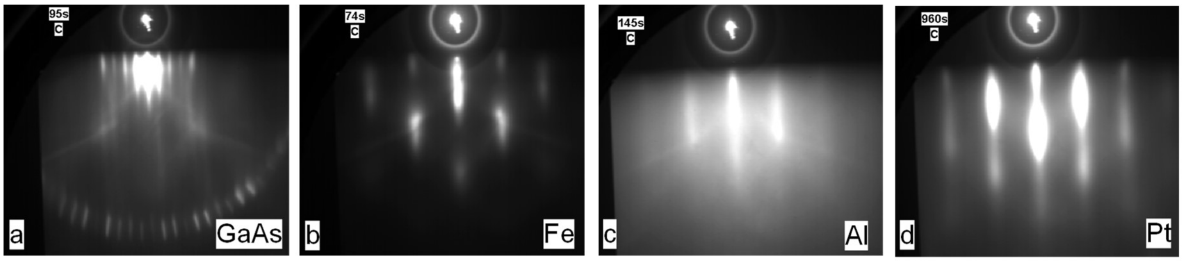

Fig. S1. RHEED images taken after the growth of GaAs (**a**), Fe (**b**), Al (**c**) and Pt (**d**).

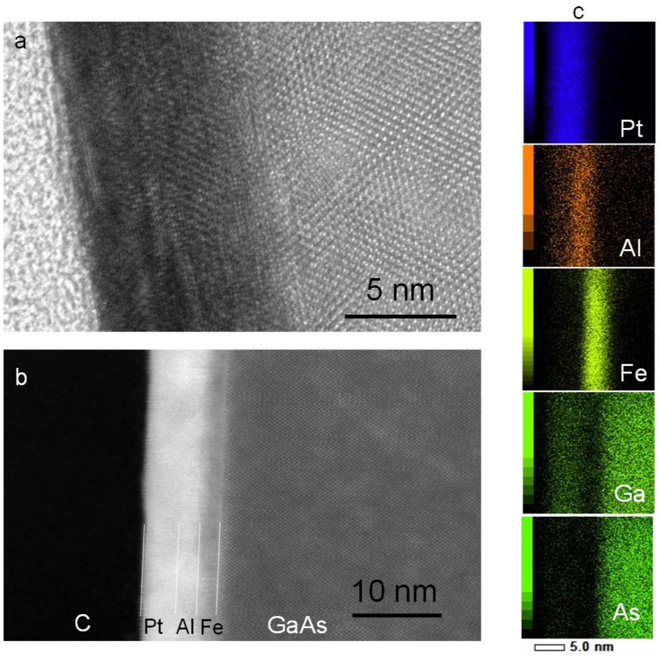

Fig. S2. (**a**) HRTEM image of the Pt/Al/Fe/GaAs multilayer. (**b**) HAADF-STEM image. (**c**) EDX of each element.

Supplementary Note 1: Characterization of the Pt/Al/Fe/GaAs multilayers

Figs. S1a-d show, respectively, the reflection high-energy electron diffraction (RHEED) images of GaAs, Fe, Al and Pt. Sharp streaks have been observed after the growth of each layer by molecular beam epitaxy at room temperature, which indicate the epitaxial growth mode as well as good surface

(interface) flatness.

To further characterize the property of our samples, we have carried out high resolution transmission electron microscopy (HRTEM) measurements. Before the HRTEM measurements, sample has been prebaked at 110 ºC, which is the highest temperature for device fabrication. The HRTEM image shows (Fig. S2a) that all the layers are crystalline, due to the pseudomorphic growth (as indicated by RHEED patterns shown in Fig. S1). To resolve the distribution of the atoms, the *Z*-contrast in the high-angle annular dark field (HAADF) scanning transmission electron microscopy (STEM) image is shown in Fig. S2b, and blurry boundaries (white lines) for each layer can still be seen. The Pt, Al, Fe, Ga and As elemental chemical maps (Fig. S2c) have also been acquired using energy-dispersive X-ray (EDX) spectroscopy, which shows that there is no significant diffusion of Pt atoms into Fe. Note that a tiny amount of Ga is also present in the Pt layer, which is generated by the cut of sample into lamina by focused-ion-beam. The good crystallinity of the Al layer prevents the diffusion of Pt towards Fe, and therefore the magnetic proximity effect can be largely blocked (more discussions about the magnetic proximity effect are shown in Supplementary Note 3). Although a diffusion of Al into Pt is observed in Fig. S2c, a sizeable spin-torque efficiency of Pt-Al alloy (Methods) is still obtained. Previous work has shown that the spin-torque efficiency can be enhanced by alloying Pt with Al[S1].

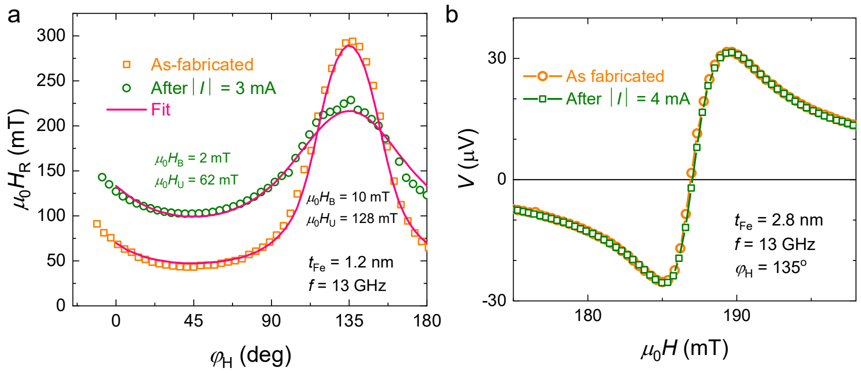

Fig. S3. (**a**) φ_H-dependence of H_R of t_{Fe} = 1.2 nm measured in the as-fabricated state and after applying |*I*| of 3 mA. The application of the relatively large current partially destroys the magnetic properties, i.e., the uniaxial anisotropy decreases from 128 mT to 62 mT and the biaxial anisotropy decreases from 10 mT to 2 mT. (**b**) FMR spectra measured in the as-fabricated state and after applying a dc current |*I*| of 4 mA for t_{Fe} of 2.8 nm. The FMR spectrum remains unchanged after the application of the dc current.

t_{Fe} (nm)	4.5	2.8	2.2	1.2	1.1
$\lvert I_{opt}\rvert$ (mA)	4.5	4	4	1.5	1.5

Table S1. Optimal current applied for each Fe thickness without detrimental effect by Joule heating.

Supplementary Note 2: Ruling out possible detrimental effects arising from the application of a dc current

To observe the modification of magnetic anisotropies and Gilbert damping, sizeable charge currents are needed. However, the application of charge currents inevitably generates Joule heating, and could partially and/or completely damage the device. Figure S3a shows an example for the case that applying a relatively large current of 3 mA significantly reduces the uniaxial magnetic anisotropy from 128 mT to 62 mT, and reduces the biaxial anisotropy from 10 mT to 2 mT for t_{Fe} = 1.2 nm. In this case, the change of magnetic properties is irreversible since the device is partially damaged probably due to interdiffusion. To rule out the possibility that the current-induced modification is not

due to any detrimental effect by Joule heating, we increase the magnitude of the current step-by-step, and measured the FMR spectra before and after applying the dc current. An optimal current value $|I_{opt}|$ is determined by two criteria: I) it shows sizeable modification of the linewidth and the resonance field, and II) the FMR spectra should be identical before and after applying the optimal charge current. An example is shown in Fig. S3b for t_{Fe} = 2.8 nm. According to our expertise, although the device sizes (4 μm × 20 μm) and the Pt (6 nm)/Al (1.5 nm) layer thicknesses are identical, the optimal current for each t_{Fe} differs. As summarized in Table S1, the thinner the t_{Fe}, the lower the $|I_{opt}|$ is.

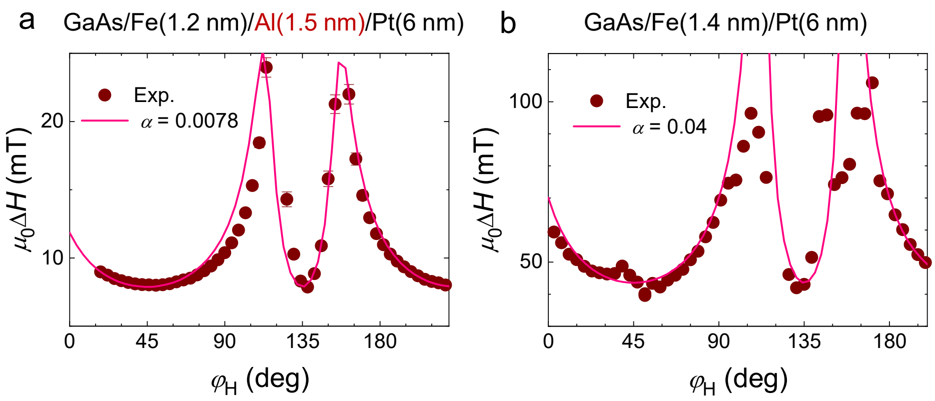

Figure S4 (a) φ_H-dependence of ΔH for GaAs/Fe(1.2 nm)/Al(1.5 nm)/Pt(6 nm) measured at f = 13 GHz. (b) φ_H-dependence of ΔH for GaAs/Fe(1.4 nm)/Pt(6 nm) measured at f = 18 GHz. The solid lines in (a) and (b) are fits using the corresponding damping values as indicated. The sample without Al separation layer shows a ~5 times larger damping than the sample with Al.

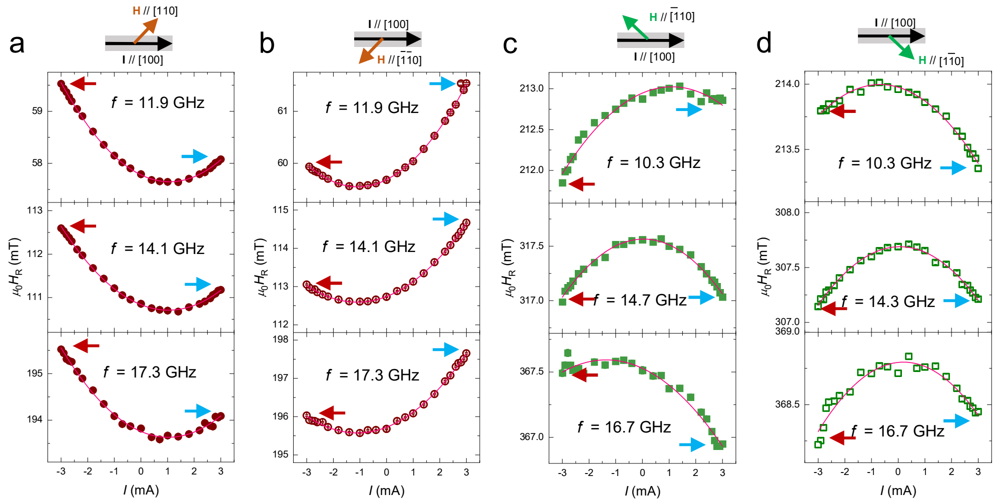

Fig. S5. *I*-dependence of H_R of a Pt(6 nm)/Cu(2 nm)/Fe(1.3 nm)/GaAs multilayer measured at selected frequencies for *H* along the [110]-axis (**a**), the $[\bar{1}\bar{1}0]$-axis (**b**) the $[\bar{1}10]$-axis (**c**) and the $[1\bar{1}0]$-axis (**d**). The inset in each figure shows the respective orientation of the charge current and the magnetic-field (magnetization). The solid lines are fitted by eq. 2, and the corresponding $d(H_R)/dI$ values are obtained. When *H* is applied along the easy axis ([110] and $[\bar{1}\bar{1}0]$), the relative amplitude of $H_R(-I)$ and $H_R(+I)$ is independent of *f*. However, when *H* is applied along the hard axis ($[\bar{1}10]$ and $[1\bar{1}0]$), the relative amplitude of $H_R(-I)$ and $H_R(+I)$ strongly depends on *f*.

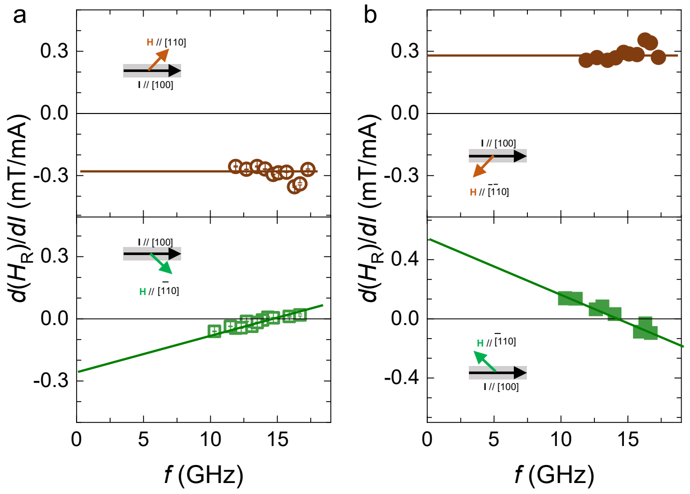

Fig. S6. Results obtained for a Pt(6 nm)/Cu(2 nm)/Fe(1.3 nm)/GaAs multilayer: (a) *d*(H_R)/*dI* values as a function of *f* for **H** // **M** // [110] (easy axis) and **H** // **M** // $[1\bar{1}0]$ (hard axis). (**b**) *d*(H_R)/*dI* values as a function of *f* for **H** // **M** // $[\bar{1}\bar{1}0]$ (easy axis) and **H** // **M** // $[\bar{1}10]$ (hard axis). The inset in each figure shows the orientation of **H** with respect to the current.

	Pt/Cu/Fe/GaAs	
	+**M**	−**M**
ΔH_B (mT)	0.28	−0.36
ΔH_K (mT)	1.12	−1.63
ΔH_U (mT)	1.48	−1.92
$\lvert h_{Oe/FL} \rvert$ (mT)	1.52	

Table S2. Summary of ΔH_B, ΔH_K, ΔH_U and $h_{Oe/FL}$ for Pt(6 nm)/Cu(2 nm)/Fe(1.3 nm)/GaAs (001) multilayer under I = 1 mA.

Supplementary Note 3: Discussion of the effect of the magnetic proximity effect

In Pt/ferromagnet bi-layers without separation layer, the magnetic proximity seems inevitable because Pt is in the vicinity of the Stoner condition. The static and dynamic exchange coupling between the ferromagnet and the proximity magnetized Pt layer produces two effects which can be discerned in the FMR measurements[S2]: I) Static coupling, which is proportional to **M**, generates a torque acting on the ferromagnet and manifests itself as a shift of H_R. II) Dynamic coupling, which is proportional to $d\mathbf{M}/dt$, plays a role for the enhancement of damping.

To show that the magnetic proximity effect can be largely avoided in the Pt/Al/Fe/GaAs samples, we have prepared and measured Pt/Fe/GaAs samples without Al separation layer. As shown in Fig. S4, the GaAs/Fe/Pt sample without Al layer (Fig. S4b) shows a ~ 5 times larger damping value than the GaAs/Fe/Al/Pt sample with Al layer (Fig. S4a). The much lower damping value in the GaAs/Fe/Al/Pt sample indicates that the Al insertion layer significantly reduces the magnetic proximity. Note that the damping value of the GaAs/Fe/Al/Pt sample is just slightly higher than that of a pure GaAs/Fe/AlO_x sample (Extended Data Fig. 3) due to the spin pumping effect. Therefore, we conclude that the Al separation layer indeed plays a significant role in minimizing the magnetic

proximity effect.

To further prove that the magnetic proximity effect is irrelevant for the modification of magnetism by spin current, we have also measured a Pt(6 nm)/Cu(2 nm)/Fe(1.3 nm)/GaAs multilayer with Cu separation layer. This is because the Cu interlayer significantly reduces the magnetic proximity effect[S2-S5]. It has also been shown[S6] that a 1-nm Cu separation layer is enough to eliminate the magnetic proximity effect as evidenced by XMCD. Figures S5a and b respectively show the I-dependence of H_R measured at selected frequencies for H along the [110]-axis and the $[\overline{11}0]$-axis. One can see that $H_R(-I) > H_R(+I)$ for **H** // [110] and $H_R(-I) < H_R(+I)$ for **H** // $[\overline{11}0]$. For both [110]- and $[\overline{11}0]$-orientations, the relative amplitude of $H_R(-I)$ and $H_R(+I)$ is independent of frequency. However, for **H** along the $[\bar{1}10]$-axis and the $[1\bar{1}0]$-axis as shown in Figures S5c and d, the relative amplitude of $H_R(-I)$ and $H_R(+I)$ strongly depends on frequency, similar to the results presented in Fig. 3 of the main text.

The I-dependence of the H_R traces are fitted by equation (2), and the f-dependence of the $d(H_R)/dI$ values is shown for each field orientation in Fig. S6. The sample with Cu separation shows the same results as the sample with Al separation, and the modification of the magnetic anisotropies (Table S2) quantitatively matches the results with Al separation (Table 2). Therefore, we confirm that the observation of the modification of magnetism by spin current is not related to the magnetic proximity effect.

Although the magnetic proximity effect has been largely avoided in our samples by inserting Al

and Cu separation layers, we cannot completely exclude this effect. We would like to point out, however, that the magnetic proximity effect (if it exists) plays no role for the modification of magnetism by spin current. This is because the modification of magnetism is only related to the damping-like torque. It has been shown (e.g., Ref. S7) that the magnetic proximity effect in heavy metal/ferromagnetic metal bilayers has no discernible influence on the magnitude of the current-induced spin-orbit torques, spin memory loss and spin backflow. Therefore, we confirm that the magnetic proximity effect does not affect the physics discussed in the work.

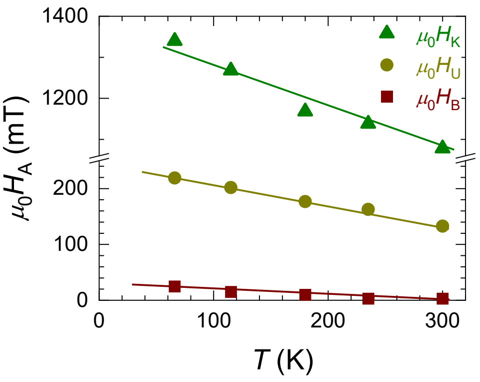

Fig. S7. Temperature dependence of H_K, H_U and H_B for t_{Fe} = 1.2 nm.

Supplementary Note 4: Theoretical calculation of the modification of magnetic moment and magnetic anisotropy

As the magnetic anisotropy plays a central role in this study, it is useful to clarify the technique used to calculate the energy of the magneto-crystalline anisotropy (MCA) from first principles. We will focus here on the spin-orbit induced MCA contribution only, because of its responsibility for the in-plane uniaxial magnetic anisotropy (UMA) observed in the Fe/GaAs(001) system[S8]. In the present

work, the MCA energy is obtained by making use of the magnetic torque calculated by means of the fully relativistic KKR Green function formalism (see e.g. Refs. S9 and S10, and more references can be found in S10). This approach is especially convenient when dealing with systems with uniaxial anisotropy, as it gives direct access to the uniaxial magnetic anisotropy energy[S11]. In the case considered here, the electronic structure is calculated for the system with the magnetic moment oriented along the *z*-axis ($\mathbf{z} \parallel [001]$). Using these results, the magnetic torque is calculated for the magnetic moment rotated by an angle of $\pi/4$ with respect to the *z*-axis within the plane including the [001] and [110] crystallographic directions. For such a geometry the calculated torque is equal to the energy difference $T_{[110]}(\pi/4) = E_{[110]} - E_{[001]}$, where $E_{[110]}$ and $E_{[001]}$ are the total energies for the system with the magnetic moment along the [110] and [001] directions, respectively. Similarly, the magnetic torque $T_{[1\text{-}10]}(\pi/4)$ gives access to the energy difference $E_{[1\text{-}10]} - E_{[001]}$, i.e. $T_{[1\text{-}10]}(\pi/4) = E_{[1\text{-}10]} - E_{[001]}$. Therefore, the in-plane uniaxial magnetic anisotropy, which is of central interest here, can be obtained from the torque difference, i.e., $E_{[1\text{-}10]} - E_{[110]} = T_{[1\text{-}10]}(\pi/4) - T_{[110]}(\pi/4)$. Furthermore, one has to stress that the in-plane uniaxial anisotropy is determined first of all by the atomic geometry at the Fe/GaAs interface. However, the absolute value of the anisotropy energy may be tuned by changing the spin magnetic moment of the atoms, for example, by electron spin transmission into the FM, as suggested in the present work. Alternatively, the spin magnetic moment of the Fe atoms may be changed by applying a magnetic field along the direction of the magnetic moment (i.e. along the quantization axis parallel to the *z*-axis), or opposite to it. Then, the resulting self-consistent potential is used to calculate the in-plane uniaxial magnetic anisotropy (modified due to the applied magnetic field) by means of the magnetic torque calculations as described above. This way, the dependence of the in-plane uniaxial magnetic anisotropy on the magnetic moment of the Fe atoms has been investigated by varying the strength of the magnetic field.

To supply theoretical support to the discussions on the impact of a spin current on the magnetic anisotropy in Fe films on GaAs(001), we are going to use rather simple arguments, which allow to explain the experimental results in terms of the electronic structure. Coming back to the idea represented at the beginning in the main text, we discuss the spin current $\mathbf{J}_S^z$ through the Pt/Al/Fe interface, polarized parallel to the Fe magnetization **m** in the case of **m** // +**z** , or antiparallel, in the case of **m** // –**z**. The spin current $\mathbf{J}_S^z$ can be represented in terms of two opposite flows of electrons carrying up and down spin moments, $\mathbf{J}_S^z = \mathbf{J}_{up} - \mathbf{J}_{dw}$, in the absence of a net electric current ($\mathbf{J}_c = \mathbf{J}_{up} + \mathbf{J}_{dw} = 0$). As a consequence, the inflow of the spin-up electrons leads to an increase of occupation of the spin-up d-states of Fe, while the outflow of spin-down electrons leads to a decrease of occupation of the spin-down d-states. Therefore, this leads to an increase of the spin-up electron density Δn_{up} and decrease of spin-down electron density Δn_{dw}, resulting in the change of magnetization $\Delta\mathbf{m}_{tr}$ ($\Delta\mathbf{m}_{tr} = \Delta n_{up} - \Delta n_{dw}$).

A similar effect occurs in the presence of an applied magnetic field on the scale of molecular field **H** // +**z** shifting the spin-up d-states down in energy, followed by an increase of their occupation, and vice versa, the spin-down d-states shifting up in energy, leading to their depopulation. As a result, the magnetic moments of the Fe atoms increase, if their direction is parallel to the applied field. And decrease of magnetic moment is expected in the case of an antiparallel orientation with respect to the magnetic field. The dependence of the magnetic moment on an applied magnetic field can be studied within first-principles SDFT calculations. Using a varying strength of magnetic field, one can model the impact of the spin current injected into the Fe film on the magnetic properties of the film, depending on the density of the spin current. It should be noted, however, that the strength of the

magnetic field used in the calculations is the same for all layers in the Fe film and does not depend on the distance from the Pt/Fe interface. This is in contrast to the real case that an injected spin current decays in the Fe film with the distance from the interface. Nevertheless, even in the case of a uniform strength of the applied magnetic field, the magnetic response in the Fe film is not trivial, leading to different changes of magnetic moments in the various layers, Δm_i (see in Fig. S8a the results for 9 MLs of Fe deposited on GaAs(001)). This is, in particular, a consequence of their different spin- and layer-resolved density of states at the Fermi level.

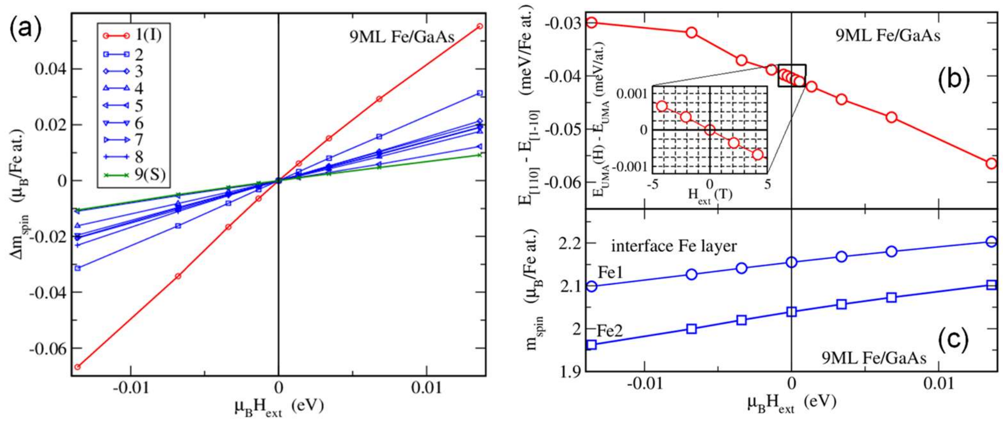

Fig. S8. Calculated results for 9ML Fe films deposited on GaAs(001). (**a**) The layer-resolved change of magnetic moments as a function of applied magnetic field, the 1st layer (I) corresponds to the Fe/GaAs interface, and the 9th layer (S) is the surface layer. (**b**) The energy of the in-plane uniaxial MCA as a function of applied magnetic field. The inset shows a zoom of the selected part of graph, which is used to quantify the effective magnetic-field values for the modification of UMA. (**c**) Spin magnetic moment of two non-equivalent Fe atoms at the Fe/GaAs interface as a function of the applied magnetic field. Here positive (negative) value corresponds to magnetic field parallel (antiparallel) to the magnetization direction.

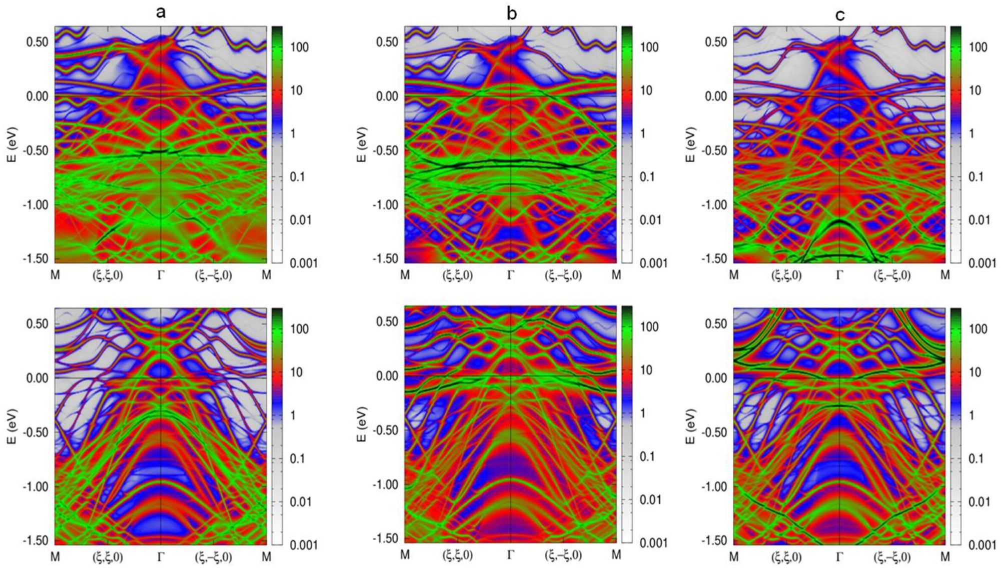

Fig. S9. Layer resolved spin-up (top panel) and spin-down (bottom panel) Bloch spectral function (BSF) for 9ML Fe films deposited on GaAs(001): (**a**) interface Fe layer, (**b**) middle Fe layer, and (**c**) surface Fe layer.

As a next step, we focus on the dependence of the MCA energy on the applied magnetic field, which is used to mimic the effect of an injected spin-current. These calculations are performed for 9 MLs of Fe deposited on GaAs(001) (we consider here the As-terminated interface) (Fig. S8b). As the MCA depends on the electronic structure in the vicinity of the Fermi energy, Fig. S9 represents the spin-resolved Bloch spectral function (BSF) $A(E, \mathbf{k})$ within a small energy window around E_F, for the wave vector **k** along two high-symmetric directions in the 2D Brillouin zone, i.e., $\mathbf{k} = (\xi, \xi, 0)$ and $(\xi, -\xi, 0)$. The intensity of the BSF in Fig. S9 characterize the degree of localization of the electronic states in the interface Fe layer (Fig. S9a), in central layer of the film (Fig. S9b) and at the surface (Fig. S9c), respectively. One can see the obvious difference of the BSF for these two directions. The symmetry of the deposited Fe film is governed by the two-fold symmetry of the GaAs(001) substrate. As one can see that the most distinct asymmetry for the electronic structure along the $(\xi, \xi, 0)$ and $(\xi, -\xi, 0)$ directions is observed for the interface Fe layer due to a strong hybridization of the electronic

states of Fe with the states of GaAs. Furthermore, a significant smearing of the energy bands is observed for the interface Fe layer, which are hybridized with bulk-like states of the GaAs substrate. In the middle and surface Fe layers, the smearing of the d-bands, as well as their asymmetry along two BZ directions, decreases. The symmetry properties of the electronic structure determine the symmetry properties of the magnetic anisotropy, leading to the interfacial origin of uniaxial in-plane MCA.

Fig. S8b shows corresponding field-induced changes of the energy of in-plane uniaxial MCA, $E_{UMA} = E_{[110]} - E_{[1\text{-}10]}$. The energy is represented in meV per one Fe atom. As one can see that, in the absence of magnetic field, the easy axis is oriented along the [110] crystallographic direction. The applied magnetic field results in an increase of the MCA energy along the easy axis, E_{UMA}, if it is parallel to the magnetization direction, and to a decrease of E_{UMA} in the case of antiparallel orientation. These changes are accompanied by an increase (for $H > 0$) or decrease (for $H < 0$) of the spin magnetic moment of Fe, which is shown in Fig. S8a (the layer-resolved Δm_i), and in Fig. S8c (the magnetic moments for the interface Fe atoms).

In order to make a connection between the theoretical and experimental results, one can estimate the strength of the magnetic field to be used in the calculations to mimic the role of the spin current, which induces the change of the in-plane uniaxial magnetic anisotropy energy ΔE_{UMA}. In the experiments, the application of a charge current of ~1 mA in the Pt layer induces a change of the in-plane UMA characterized by the anisotropy magnetic field $\Delta H_U \approx 2$ mT. Using the 'average' value of 2.25 μ_B for the magnetic moment per Fe atom, one obtains the change of in-plane UMA energy,

ΔE_{UMA}, of about $2.6{\times}10^{-4}$ meV/Fe atom. To connect our theoretical results to the experiment, the inset in Fig. S8b plots the dependence of the in-plane UMA energy induced by the external magnetic field H_{ext}, applied parallel or antiparallel to the magnetization direction, $E_{\mathrm{UMA}}(H) - E_{\mathrm{UMA}}(H{=}0)$. Using these results, a magnetic field of about 1.5 T needs to be applied to obtain the same change of the in-plane UMA energy as observed in the experiments.

To further compare of the theoretical results with experiment, we represent the magnetic field (shown in Fig. S8 in the units of energy) in terms the spin moment transferred into the Fe film, which was estimated by equating the field induced change of the total spin magnetic moment in the Fe film Δm_{Fe} (shown in Fig. S8c) with the spin moment transferred due to a spin current through the interface, $\Delta m_{\mathrm{Fe}} = \Delta m_{\mathrm{tr}} = \Delta m_{\mathrm{up}} - \Delta m_{\mathrm{dw}}$. Taking into account the zero electric current ($J_c = 0$) through the interface, one can make a connection between the absolute values of the transferred spin-up density and corresponding change of the magnetization $|\Delta \mathrm{m}_{\mathrm{tr}}| = 2|\Delta n_{\mathrm{up}}|$.

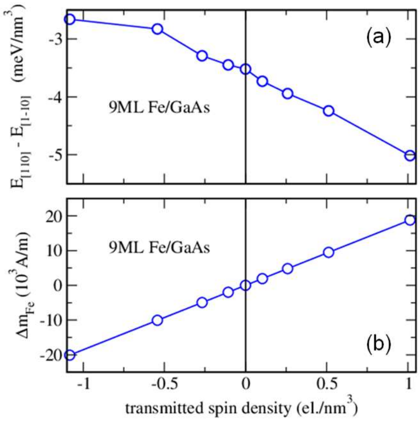

Fig. S10. (**a**) The MCA energy density as a function of the transferred spin density for 9 ML Fe films deposited on GaAs(001). The 'plus' sign corresponds to a transferred spin moment parallel to the magnetization direction, while the 'minus' sign corresponds to the opposite orientation of the transferred spin moment. (**b**) The dependence of the modified magnetization of 9ML Fe films as a function of transferred spin density.

As a result, Fig. S10 replots Fig. S8, which shows the MCA energy density as a function of transferred electron density. By converting the MCA energy density into effective UMA magnetic anisotropy field, one obtains a modulation amplitude of -228 mT/(el./nm^3). Considering the experimentally observed ΔH_U of 2.5 mT, we estimate the number of transferred spin density to be 0.01 el./nm^3 (i.e., ~10^{19} cm^{-3}). In addition, Fig. S10b shows the dependence of the modified Fe magnetization as a function of the transferred spin density.

Finally, Fig. S11 represents a polar plot for the 9ML Fe/GaAs(001) system, showing the dependence of the MCA energy density, $E(M_{||}(\varphi)) - E_{[1\text{-}10]}$, on the magnetization angle φ. The results are obtained for two values of the magnetic field corresponding to a transferred spin density of 1.05 el./nm^3 (curve 1) and 1.3 el./nm^3 (curve 2), respectively, demonstrating that the impact of the transferred spin moment on the MCA energy density is anisotropic with 2-fold symmetry.

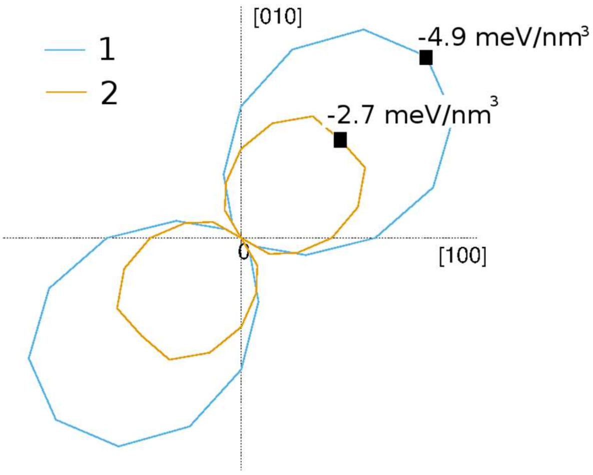

Fig. S11. Results for a 9 ML Fe film deposited on GaAs(001): polar plot for the MCA energy density, $E(M_{||}(\varphi))$ - $E_{[1\text{-}10]}$, as a function of the magnetization angle φ. Line '1' corresponds to a transferred spin-up electron density of 1.05 el./nm^3, and line '2' represents the results for a transferred spin-up electron density of -1.3 el/nm^3.

References

S1. Nguyen, M. *et al*. Enhancing spin Hall torque efficiency in $Pt_{100-x}Al_x$ and $Pt_{100-x}Hf_x$ alloys arising from the intrinsic spin Hall effect, *Appl. Phys. Lett.* **108**, 242407 (2016).

S2. Sun, Y. *et al*. Damping in Yittrium Iron Garnet nanoscale films capped by Platinum, *Phys. Rev. Lett*. **111**, 106601 (2013).

S3. Nakayama, H. *et al*. Spin Hall magnetoresistance induced by a nonequilibrium proximity effect, *Phys. Rev. Lett*. **110**, 206601 (2013).

S4. Du, C. *et al*. Enhancement of pure spin current in spin pumping $Y_3Fe_5O_{12}$/Cu/metal trilayers through spin conductance matching, *Phys. Rev. Appl*. **1**, 044004 (2014).

S5. Amanou, W. *et al*. Magnetic proximity effect in Pt/$CoFe_2O_4$ bilayers, *Phys. Rev. Mater*. **2**, 011401(R) (2018).

S6. Caminale, M. *et al*. Spin pumping damping and magnetic proximity effect in Pd and Pt spin-sink layers, *Phys. Rev. B*. **94**, 014414 (2016).

S7. Zhu, L. J. *et al*. Irrelevance of magnetic proximity effect to spin-orbit torques in heavy-metal/ferromagnet bilayers, *Phys. Rev. B* **98**, 134406 (2018).

S8. Košuth, M. *et al*. Magnetic anisotropy of thin Fe films on GaAs. *Europhys. Lett.* **72**, 816-822 (2005).

S9. Ebert. H. *et al*. The Munich SPR-KKR package, version 8.5, https://www.ebert.cup.uni-muenchen.de/en/software-en/13-sprkkr

S10. Mankovsky, S. and Ebert, H., First-principles calculation of the parameters used by atomistic magnetic simulations, *Electronic Structure* **4**, 034004 (2022).

S11. Wang, X. *et al*. Torque method for the theoretical determination of magnetocrystalline

anisotropy, *Phys. Rev. B* **54**, 61 (1996).
